# Prognostic and Clinicopathological Significance of Downregulated p16 Expression in Patients with Bladder Cancer: A Systematic Review and Meta-Analysis

**DOI:** 10.1155/2016/5259602

**Published:** 2016-04-20

**Authors:** Xiaoning Gan, Xiaomiao Lin, Rongquan He, Xinggu Lin, Hanlin Wang, Liyan Yan, Hong Zhou, Hui Qin, Gang Chen

**Affiliations:** ^1^Department of Pathology, First Affiliated Hospital of Guangxi Medical University, Nanning, Guangxi Zhuang Autonomous Region 530021, China; ^2^Department of Children Rehabilitation Medicine, Guangxi Maternal and Child Health Hospital, 225 Xinyang Road, Nanning, Guangxi Zhuang Autonomous Region 530003, China; ^3^Center of Genomic and Personalized Medicine, Guangxi Medical University, Nanning, Guangxi Zhuang Autonomous Region 530021, China

## Abstract

p16, encoded by the* CDKN2A* gene, is a tumor suppressor that has been widely studied in cancer research. However, the relationship of p16 with prognostic and clinicopathological parameters in patients with bladder cancer remains unclear. Data inclusion criteria were articles reporting on the relationship between p16 expression and the prognosis or clinicopathology in patients with bladder cancer. Meta-analyses were performed with Stata software. Hazard ratios (HRs) or odds ratios (ORs) and 95% confidence intervals (95% CI) were calculated to evaluate the relative risks. The source of heterogeneity was analyzed by subgroup analysis. A total of 37 studies with 2246 cases were included and analyzed. The results identified an important link between downregulated p16 expression and poor prognosis in patients with bladder cancer in terms of recurrence-free survival (RFS), overall survival (OS), progression-free survival (PFS), and some clinicopathological parameters including clinical staging, pathological degree, and lymph node metastasis. Subgroup analysis also showed that low p16 expression could function as a warning sign for RFS and PFS in patients with early-stage (Ta–T1) bladder cancer. In conclusion, p16 might play an essential role in the deterioration of bladder cancer and could serve as a biomarker for the prediction for patients' progression and prognosis.

## 1. Introduction

Bladder cancer is the most frequent malignancy of the urinary tract and the ninth most common cancer worldwide [1]. About 95% of bladder cancers are histologically transitional cell carcinoma, with rare cases of squamous cell carcinoma and adenocarcinoma. However, the pathogenesis of bladder cancer is still unclear, and its occurrence and development appear to be affected by multiple genes [2]. Serrano et al. first cloned the cDNA of the gene encoding the tumor suppressor protein p16 (CDNK2A) in 1993; since then it has been widely studied in the field of cancer research [3]. Previous studies have reported ubiquitous downregulation of p16 gene expression in bladder cancer, as a result of various alterations including complete deletion, point mutation, or promoter methylation [4–6]. Furthermore, p16 could compete with cyclin D1 for binding to Cyclin Dependent Kinase (CDK) 4/6, thus blocking the phosphorylation of retinoblastoma (Rb) protein and inhibiting release of the transcription factor E2F, preventing cell conversion from G_1_ phase to S phase, and eventually suppressing cell proliferation. These results suggest that abnormal expression of the p16 gene in cells might be associated with tumorigenesis [6, 7].

**Figure 1:**
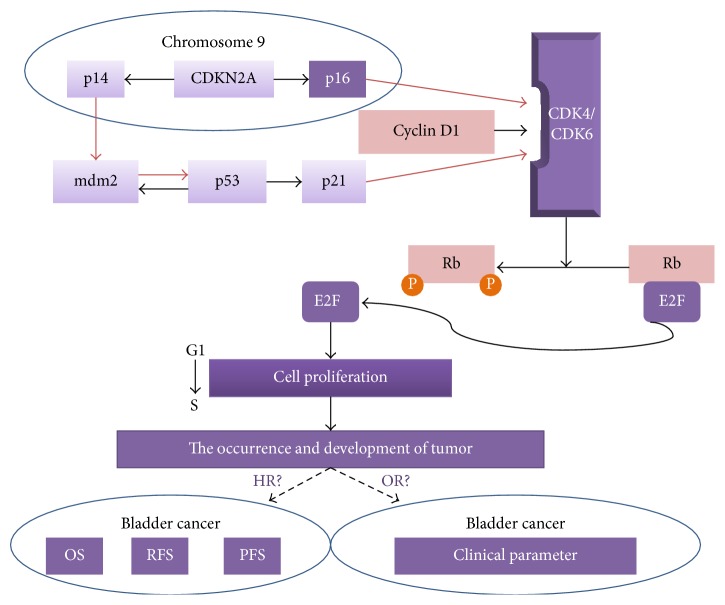
Main molecular pathways of bladder cancer (adapted from Mitra et al. [7]).

Numerous studies to date have explored the clinicopathological and prognostic significance of p16 in patients with bladder cancer. However, as a result of differences in sample sizes, accuracies of the statistical data, study populations, and interventions, the results remain inconclusive, and evidence-based confirmation by large-scale clinical trials is still lacking. We therefore conducted an in-depth systematic review and meta-analysis to investigate the correlation between abnormal expression of p16 and clinicopathological features, as well as prognosis in patients with bladder cancer. The specific mechanisms are shown in Figure 1.

## 2. Materials and Methods

### 2.1. Literature Search

The terms and combinations including “Cyclin Dependent Kinase Inhibitor p16,” “CDKN2A Protein,” “p16INK4A Protein,” “MTS1 Protein,” “Cyclin Dependent Kinase Inhibitor 2A,” “Multiple Tumor Suppressor 1,” “Cdk4 Associated Protein p16,” “TP16,” and “urinary bladder neoplasms,” “bladder tumors,” “bladder cancers,” “bladder carcinomas,” and “prognos^*∗*^,” “surviv^*∗*^,” “follow-up,” “mortality,” “predict,” “course,” “outcome,” and “clinicopathological” were used to search the following domestic and international databases: PubMed, Wiley Online Library, Embase, Cochrane Central Register of Controlled Trials, Science Direct, EBSCO, Google Scholar, Ovid, LILACS, China National Knowledge Infrastructure (CNKI), China Biology Medicine disc (CBMdisc), CQVIP, and Wan Fang, with unified retrieval rules such as Boolean logic. The obtained search results were then analyzed, evaluated, reviewed, and manually screened to determine their relevance.

### 2.2. Inclusion and Exclusion Criteria

Inclusion criteria were as follows: (1) patients diagnosed with bladder cancer; (2) immunohistochemical (IHC) detection of p16 expression levels in the tissues; (3) relationships between abnormal expression of p16 and prognostic indicators such as recurrence-free survival (RFS), progression-free survival (PFS), and overall survival (OS) or associations between p16 and clinicopathological features that were evaluated; (4) hazard ratio (HR), odds ratio (OR), relative risk (RR), and 95% confidence intervals (CI) that could be obtained directly from the full article or indirectly calculated with relevant software based on the data provided in the graphics and tables; (5) only the newest studies or the ones with higher quality were retained if the data were repeated in different studies; and (6) studies in English or Chinese.

Exclusion criteria were as follows: (1) cell or animal studies, case reports, letters, reviews, and meta-analyses; (2) articles with similar content or using the same data or those with small sample sizes (*n* ≤ 10) and those with no directly or indirectly extractable HR, OR, and 95% CI data; and (3) articles that could not be understood because of language barriers.

### 2.3. Data Extraction

Two independent investigators (Xiaoning Gan and Rongquan He) reviewed the articles that met the criteria and extracted data on author, year of publication, nationality, sample size, patient age, detection method of p16, antibody source and dilution, clinical stage, pathological degree, other costudied prognosis-associated genes, cut-off value, outcome, and extraction method of the study subjects. Discrepancies between the two independent investigators in terms of data extraction were resolved by discussion among all the authors.

### 2.4. Statistical Analysis

Effects of p16 on the related prognostic indexes were detected by merging the HRs and 95% CI of the included literatures, which were evaluated through the Forest plot and related parameters after the merging. The HRs and 95% CI values mainly came from direct extraction of the original text or survival curve through extraction and calculation by software.

The relationships between p16 and the clinicopathological parameters were derived from the binary variable data extracted from the original articles. ORs and 95% CI values came from the binary variable data calculated by Stata software. The data were then combined, and their statistical significance was evaluated by Forest plot and related parameters, to clarify the relationship between p16 low-expression and clinicopathological parameters.

Heterogeneity was measured by *Q* statistics as follows: no heterogeneity: 0 < *I* ^2^ < 25%; low heterogeneity: 25% ≤ *I* ^2^ < 50%; moderate heterogeneity: 50% ≤ *I* ^2^ < 75%; high heterogeneity: 75% ≤ *I* ^2^ ≤ 100%. If *I* ^2^ < 50% and *P* > 0.10, a fixed-effect model would be used in combination with HRs, ORs, and 95% CI; if *I* ^2^ ≥ 50% and *P* ≤ 0.10, then a random-effect model would be selected. Heterogeneity analysis was performed to assess the accuracy of the data, and subgroup and sensitivity analyses were carried out based on professional knowledge.

Publication bias was detected by Begg's funnel plot and Egger's test with Stata software. A two-sided *P* value < 0.05 was considered to indicate statistical significance. Statistical analyses were carried out with StataSE 12.0, Engauge, Photoshop CS5, and Microsoft Office 2007.

## 3. Results

### 3.1. Eligible Studies

**Figure 2:**
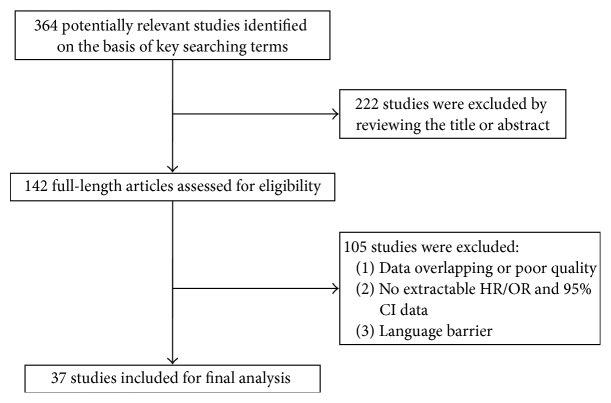
Flow diagram of studies selection procedure.

A total of 364 articles were identified from the databases, including 190 English and 174 Chinese articles, 222 of which were excluded because of discrepancies between the study theme and their abstracts. The full text of the remaining 142 articles was then reviewed for their fit with the current study, after which a further 105 articles were excluded because they met one or more of the exclusion criteria, such as the cell or animal studies, reviews, and letters and studies with identical data and no extractable HR, OR, and 95% CI data from the full text or language barrier. The remaining 37 articles [4, 5, 8–42] with 2246 cases were included in our study and consisted of 21 English [4, 5, 8–19, 21–25, 41, 42] and 16 Chinese [20, 26–40] articles. The screening process was demonstrated in Figure 2.

The basic features of the included studies were presented in Table 1. Among the 37 articles, 26 studies [4, 5, 8–21, 28–34, 39, 41, 42] investigated the relationship between low expression of p16 and prognostic parameters in bladder cancer patients (RFS, OS, PFS, and DSS/CSS), and 30 studies [4, 10–13, 15–19, 21–40] assessed the association between p16 and clinicopathological factors in patients with bladder cancer.

### 3.2. Relationship between Downregulated p16 Expression and RFS in Patients with Bladder Cancer

**Figure 3:**
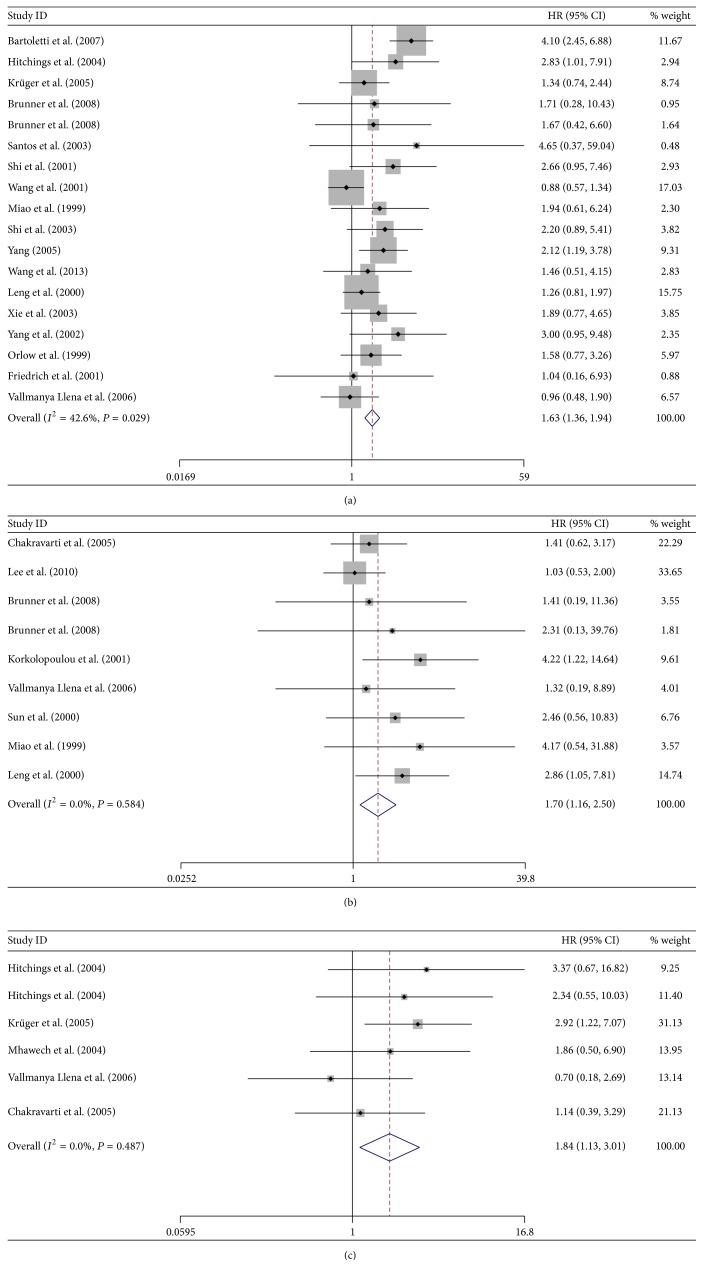
Forrest plot of hazard ratio (HR) for the association of p16 with recurrence-free survival (RFS) (a), overall survival (OS) (b), and progression-free survival (PFS) (c) in patients with bladder cancer.

A total of 17 studies with 1032 subjects were included in the final analysis of RFS [4, 5, 8, 10, 13–15, 19, 21, 28–34, 39]. Low expression of p16 was related to poor RFS in patients with bladder cancer (HR = 1.63, 95% CI = 1.36~1.94, and *P* < 0.001), with low observed heterogeneity (*I* ^2^ = 42.6%, *P* = 0.029) (Figure 3(a)).

Cumulative meta-analysis based on year of publication and sample size demonstrated that the results tended to stabilize with increasing sample size, but there was no obvious relationship between the results and year of publication.

Based on sensitivity analysis, the study by Yang et al. [13] was initially excluded because of a large difference in HR compared with the overall average, which was attributed to the selection of a different calculation method in the original article. Binary variable data were extracted and the HR and 95% CI were therefore recalculated with Stata software.

Subgroup analysis based on geographic region showed that low expression of p16 was associated with RFS in patients with bladder cancer both in Asia (HR = 1.44, 95% CI = 1.15~1.81, and *P* = 0.002) and in Europe (HR = 1.90, 95% CI = 1.13~3.19, and *P* < 0.001). The results of American studies (HR = 1.58, 95% CI = 0.77~3.25, and *P* = 0.214) need to be confirmed with larger sample sizes. The heterogeneity of Asian studies (*I* ^2^ = 30.7%, *P* = 0.173) was lower than the overall heterogeneity, while that of Europe (*I* ^2^ = 53.1%, *P* = 0.037) was higher, calculated with the random-effect model.

Subgroup analysis based on clinical stage suggested that the effect of p16 on RFS was associated with clinical stage (Tis-T1 group: HR = 1.96, 95% CI = 1.23~3.14, and *P* < 0.001; *I* ^2^ = 55.5%, *P* = 0.028; Tis–T4 group: HR = 1.41, 95% CI = 1.12~1.77, and *P* = 0.003; *I* ^2^ = 10.2%, *P* = 0.348).

Subgroup analysis based on histopathological grade showed that heterogeneity decreased from G1-G2 (HR = 4.12, 95% CI = 2.48~6.83, and *P* < 0.001; *I* ^2^ = 0%, *P* = 0.924), G1–G3 (HR = 1.44, 95% CI = 1.18~1.75, and *P* < 0.001; *I* ^2^ = 11.9%, *P* = 0.323), and G2-G3 (HR = 1.37, 95% CI = 0.78~2.42, and *P* = 0.273; *I* ^2^ = 0%, *P* = 0.802), indicating that the effects of p16 on RFS in patients with bladder cancer were closely associated with pathological grade.

Subgroup analysis also showed an effect of cut-off value on the influence of p16 on RFS (cut-off value ≤ 10%: HR = 1.83, 95% CI = 1.34~2.51, and *P* < 0.001; *I* ^2^ = 54.8%, *P* = 0.009; cut-off > 10%: HR = 1.34, 95% CI = 0.86~2.09, and *P* = 0.003; *I* ^2^ = 10.2%, *P* = 0.348).

In addition, subgroup analysis of early-stage data from 430 subjects from eight studies also demonstrated that low expression of p16 significantly affected RFS in patients with early-stage (Ta–T1) bladder cancer (HR = 1.96, 95% CI = 1.23~3.14, and *P* = 0.005; *I* ^2^ = 47.9%, *P* = 0.088).

### 3.3. Relationship between the Low Expression of p16 and OS in Patients with Bladder Cancer

A total of 425 subjects in eight studies were included in the final analysis of OS [9, 11, 14, 16, 19, 20, 30, 34], which showed that low expression of p16 was associated with decreased OS in patients with bladder cancer (HR = 1.70, 95% CI = 1.16~2.50, and *P* = 0.007), with no significant observed heterogeneity (*I* ^2^ = 0%, *P* = 0.584) (Figure 3(b)).

Cumulative meta-analysis and sensitivity analysis indicated relatively low overall heterogeneity and no study with high sensitivity.

Subgroup analysis based on geographic area showed a subtle distinction between p16 expression and OS in patients with bladder cancer in Asia (HR = 1.61, 95% CI = 0.97~2.66, and *P* = 0.065; *I* ^2^ = 0%, *P* = 0.703) and Europe (HR = 2.54, 95% CI = 1.05~6.15, and *P* = 0.039; *I* ^2^ = 27.0%, *P* = 0.250).

Subgroup analysis was also performed based on clinicopathological stages. However, limitations of sample size led to the impossibility of determining if the effects of p16 expression on OS were associated with these parameters in patients with bladder cancer (Ta–T1 group: HR = 1.57, 95% CI = 0.32~7.75, and *P* = 0.579; *I* ^2^ = 0%, *P* = 0.750; Ta–T4 group: HR = 1.59, 95% CI = 0.98~2.60, and *P* = 0.061; *I* ^2^ = 3.1%, *P* = 0.389; T2–T4 group: HR = 1.96, 95% CI = 0.99~3.88, and *P* = 0.053; *I* ^2^ = 52.1%, *P* = 0.148; low-grade group: HR = 1.41, 95% CI = 0.18~10.90, and *P* = 0.742; *I* ^2^ = 0.0%, *P* = 1.000; G1–G3 group: HR = 1.82, 95% CI = 1.16~2.84, and *P* = 0.009; *I* ^2^ = 3.9%, *P* = 0.397; high-grade group: HR = 1.41, 95% CI = 0.62–3.19, and *P* = 0.409; *I* ^2^ = 0%, *P* = 1.000).

Subgroup analysis based on cut-off value indicated that the effects of p16 on OS in patients with bladder cancer were associated with cut-off value (cut-off value ≤ 10%: HR = 1.83, 95% CI = 1.17~2.86, and *P* = 0.008; *I* ^2^ = 3.2%, *P* = 0.006; cut-off value > 10%: HR = 1.40, 95% CI = 0.66~2.96, and *P* = 0.384; *I* ^2^ = 0%, *P* = 0.951).

### 3.4. Relationship between Low Expression of p16 and PFS in Patients with Bladder Cancer

A total of 470 subjects in seven studies were included in the ultimate analysis of PFS [5, 9, 10, 12, 19, 41, 42]. The results showed a correlation between low expression of p16 and poor PFS in patients with bladder cancer (HR = 2.18, 95% CI = 1.37~3.48, and *P* = 0.001), with low heterogeneity detected (*I* ^2^ = 26.3%, *P* = 0.219).

Cumulative meta-analysis revealed no obvious characteristics because of the limited range of publication dates and the sample sizes.

Sensitivity analysis identified two studies [41, 42] as having the highest heterogeneities. Further investigation revealed that this heterogeneity was caused by different methods of measuring p16 (fluorescence in situ hybridization) and studying the influence of hemizygous or homozygous deletion of p16 on patient prognosis. These two studies were finally excluded because of their incompatible study objectives, leaving a total of 347 subjects from six studies in the final analysis of PFS. The results showed that low expression of p16 was correlated with poor PFS in patients with bladder cancer, and the heterogeneity was eliminated (HR = 1.84, 95% CI = 1.13~3.01, and *P* = 0.015; *I* ^2^ = 0%, *P* = 0.487) (Figure 3(c)).

Despite a reduced sample size, subgroup analysis of the 347 subjects from five studies [5, 9, 10, 12, 19] demonstrated that the effects of p16 expression on PFS were affected by clinical stage (Ta–T1 group: HR = 2.09, 95% CI = 1.21~3.63, and *P* = 0.002; *I* ^2^ = 0%, *P* = 0.484; T2–T4 group: HR = 1.14, 95% CI = 0.39~3.31, and *P* = 0.810; *I* ^2^ = 0%, *P* = 0.484) and geographical location (Europe: HR = 2.09, 95% CI = 1.21~3.63, and *P* = 0.002; *I* ^2^ = 0%, *P* = 0.484; America: HR = 1.14, 95% CI = 0.39~3.31, and *P* = 0.810).

Subgroup analysis based on cut-off value demonstrated some relationship between cut-off value and the influence of p16 expression on PFS (cut-off value ≤ 10%: HR = 2.61, 95% CI = 1.42~4.77, and *P* = 0.002; *I* ^2^ = 0%, *P* = 0.932; cut-off value > 10%: HR = 0.95, 95% CI = 0.41~2.18, and *P* = 0.896; *I* ^2^ = 0%, *P* = 0.579).

The results from 297 subjects with early-stage (Ta–T1) bladder cancer from four studies [5, 10, 12, 19] suggested that low expression of p16 was also significantly associated with poor PFS in early-stage bladder cancer (HR = 2.09, 95% CI = 1.21~3.63, and *P* = 0.002; *I* ^2^ = 0%, *P* = 0.484).

### 3.5. Relationship between Low Expression of p16 and DSS/CSS in Patients with Bladder Cancer

A total of 187 subjects from three studies were included in the DSS/CSS analysis [9, 17, 18]; limitation of the sample size caused the impossibility of demonstrating an association between low expression of p16 and DSS/CSS (HR = 1.52, 95% CI = 0.85~2.71, and *P* = 0.149; *I* ^2^ = 0%, *P* = 0.825).

### 3.6. Relationship between Low Expression of p16 and Clinicopathological Parameters in Patients with Bladder Cancer

The relationship between low expression of p16 and clinicopathological parameters [4, 10–13, 15–19, 21–40] was further explored by analysis of 30 studies including 1785 subjects. The results of statistical analyses were as follows: T2–T4/Ta–T1: OR = 3.13, 95% CI = 2.42~4.06, and *P* < 0.001; *I* ^2^ = 1.4%, *P* = 0.440; T1/Ta: OR = 1.55, 95% CI = 0.87~2.76, and *P* = 0.134; *I* ^2^ = 40.5%, *P* = 0.152; G3/G1-2 [43]: OR = 3.33, 95% CI = 2.51~4.42, and *P* < 0.001; *I* ^2^ = 0%, *P* = 0.519; and H/L [44]: OR = 1.20, 95% CI = 0.69~2.33, and *P* = 0.580; *I* ^2^ = 61.8%, *P* = 0.011; because of the high heterogeneity, a random-effects model was therefore applied. Meanwhile, these results demonstrated significant differences in the effects of low p16 expression in patients with bladder cancer between the two WHO clinical pathological grading methods in 1973 and 2004.

Analysis of the results for lymph node metastasis showed OR = 2.20, 95% CI = 1.26~3.83, and *P* = 0.006; *I* ^2^ = 27.2%, *P* = 0.240. The small sample size caused the impossibility of demonstrating any significant influence of pathological parameters such as muscle invasion, tumor number (multiple/single), and tumor size on the effect of p16 expression (Table 2).

### 3.7. Retrospective Review

Three studies [6, 24, 25] were retrospectively reviewed because of differences between their prognosis results and the data required by the meta-analysis. As shown in Table 3, low expression of p16 was associated with poor prognosis in patients with bladder cancer. However, some of the *P* values were <0.05 because of the small sample sizes.

### 3.8. Publication Bias

**Figure 4:**
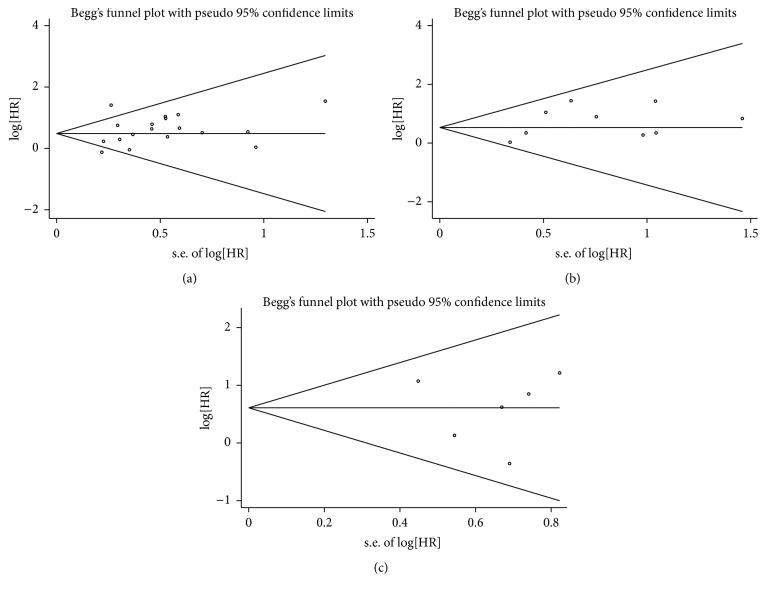
The funnel plot of the meta-analysis of the impact of p16 expression on recurrence-free survival (RFS) (a), overall survival (OS) (b), and progression-free survival (PFS) (c) in patients with bladder cancer.

Publication bias was detected by Begg's funnel plot and Egger's test (Figure 4). The points representing studies were symmetrically arranged in a funnel shape in the funnel plot, and the *P* values calculated from Egger's test with higher detection effectiveness were >0.05, indicating no publication bias. The only exception was for RFS; the funnel plot was asymmetrical and with a few points outside the funnel. Publication bias was also detected by Egger's test (G1–G3 group: *P* = 0.031; Asia group: *P* = 0.020), indicating potential publication bias in terms of RFS.

## 4. Discussion

*p16*, also known as tumor suppressor gene I (multiple tumor suppressor, MTS I), is located in 9p21 and is composed of two introns and three exons [45]. It is a key gene in cell cycle regulation, with its expression product being involved in the negative regulation of cell proliferation. Studies have shown that downregulation of p16 gene expression resulted in the loss of its inhibitory effects on CDK4/CDK6, which in turn may lead to malignant, abnormal cell proliferation and accelerated tumor development [7, 46, 47]. Elucidation of the relationship between low expression of p16 and prognosis and clinicopathology in patients with bladder cancer is therefore important for its early diagnosis, treatment, and prognosis.

Pan et al. performed a meta-analysis of the prognostic significance of abnormal p16 and p21 expression in bladder cancer in 2006 [48]. However, the current study analyzed a larger sample size; Pan et al.'s study included 12 articles with 975 cases, compared with 37 articles and 2246 cases in our study, leading to more accurate and reliable results. Secondly, Pan et al.'s study involved a number of mixed factors with no clear listing of each prognostic index or subgroup discussion. In contrast, the current study included subgroup analyses for the different indicators including RFS, PFS, OS, and DSS/CSS, allowing more thorough insights into the relationships between p16 expression and the prognostic and clinicopathological parameters in bladder cancer patients. Thirdly, Pan et al. found no association between p16 expression and prognosis in early Ta–T1 stage (stage I) bladder cancer, possibly because of the omission of the study by Krüger et al. [5], which explored the significance of p16 as an independent tumor predictive factor for the development of T1 bladder cancer, and demonstrated the important clinical value of low p16 expression in the early diagnosis and prognosis of patients with early-stage bladder cancer.

**Figure 5:**
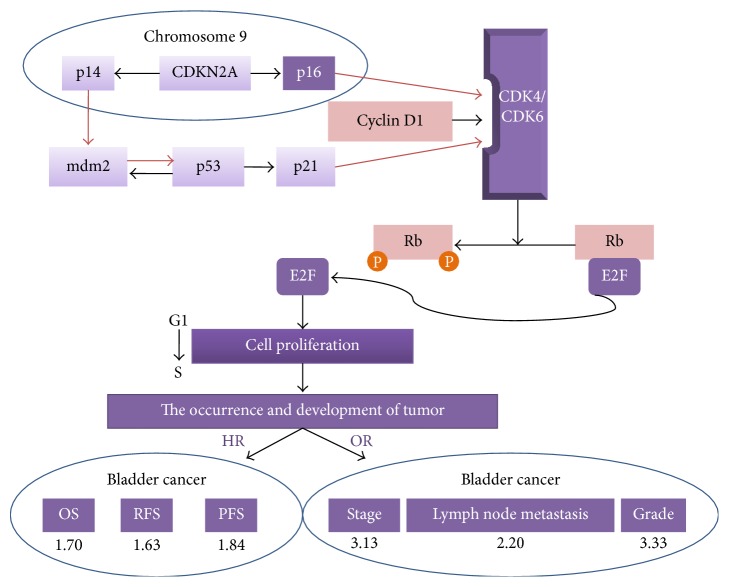
Our results illustrated and improved the relationship between p16 and prognosis, as well as clinicopathological features.

The current study systematically analyzed the relationships between p16 expression and prognostic index and clinicopathological parameters in patients with bladder cancer and showed that low expression of p16 was closely correlated with poor prognosis (Figure 5). However, the included studies varied in terms of study subjects, design, sample size, interventions, outcomes, time of study, and publication date. We used cumulative meta-analysis, sensitivity analysis, and subgroup analysis to explore the effects of the main variables in the included studies. Overall, the results confirmed that the relationship between low expression of p16 and prognosis in patients with bladder cancer was affected by clinicopathological stage, geographic origin of the study subjects, detection method, and cut-off values. Based on these findings, we further analyzed the relationships between p16 expression and clinicopathological parameters and demonstrated associations between low expression of p16 and clinical stage and lymph node metastasis, implying that the p16 gene tended to exert its regulatory effects during the early stage of bladder carcinogenesis. Low expression of p16 was also correlated with poor PFS and RFS in early-stage (Ta–T1) bladder cancer. These results thus confirmed an important role for p16 in the occurrence and development of bladder cancer. Meanwhile, through Phase I and II clinical trials, studies have revealed that CDK4/6 is an attractive target in p16 related pathway for anticancer therapy [49–51]. Furthermore, previous study also suggested that p16 functional peptide, as a molecular targeting agent, showed effective reactions for the treatment of renal cell carcinoma [52]. The results of these researches and our current meta-analysis had the effect of mutual authentication. Therefore, a better understanding of the mechanism underlying the development and progression of bladder cancer may play a significant role in prevention, target therapy, and prognosis, particularly if more sensitive and specific correlative biomarkers can be discovered and verified.

The current study had some limitations. First, tumors are the result of both environmental and genetic factors, and p16 may thus be only one of several factors involved in the whole process of bladder carcinogenesis. Secondly, heterogeneity may result from differences in intervention measures (surgery, radiotherapy, chemotherapy, or combination), immunohistochemical techniques (different antibodies, evaluation standards, etc.), and the HR extraction methods used in the included studies. Finally, the exclusion of articles because of language barriers and of studies that were not published because of a lack of sufficient data may have led to potential publication bias.

In conclusion, the results of the current study provide evidence for a relationship between p16 expression and prognosis and clinicopathological features in patients with bladder cancer. The results of this meta-analysis will help to inform about the development of clinical guidelines promoting best medical care for patients with bladder cancer. Further studies are required to investigate the combined influence of genetic and environmental factors on the development and progression of bladder cancer.

## Figures and Tables

**Table 1:** Main features of all studies included in the meta-analysis.

Author	Year	Nation	No.^b^ (M/F)	Age	Stage	Grade	Cut-off value	Outcome	Data extraction	Other costudied genes	Antibody source (dilution)	Detection method of p16
Orlow et al. [4]	1999	Canada	120	NR	Ta–T1	G1–G3	Score = 3	RFS/CP	Reported	P14	Vector (1 : 500)	Immunohistochemistry
Bartoletti et al. [8]	2007	Italy	56 (50/6)	70.1 (45–89)	Ta–T1	G1-G2	10%	RFS	Reported	9p21	Bio-Optica (1 : 25)	Immunohistochemistry
Chakravarti et al. [9]	2005	USA	50 (36/14)	NR	T2–T4	High	20%	OS/FFS/DSS	Reported	Erb-1, Erb-2, P53, PRB	Zymed (NR)	Immunohistochemistry
Hitchings et al. [10]	2004	UK	78	66 (24–90)	Ta–T1	G1–G3	10%	PFS/RFS/CP	Reported	P53, PRB	Novocastra (1 : 50)	Immunohistochemistry
Krüger et al. [5]	2005	Germany	73 (60/13)	68 (NR)	T1	G2-G3	10%	RFS/PFS	Reported	NR	Biocarta (1 : 50)	Immunohistochemistry
Lee et al. [11]	2010	Korea	47 (4/43)	NR	Ta–T4	Low and high	Score = 5	OS/CP	Reported	P53, PRB	DAKO (1 : 200)	Immunohistochemistry
Mhawech et al. [12]	2004	Switzerland	49 (44/5)	70.3 (52–90)	T1	Low and high	Score = 3	PFS/CP	Reported	P21	DAKO (1 : 20)	Immunohistochemistry
Yang et al. [13]	2002	China	67	NR	T1-T2	G1–G3	5%	RFS/CP	Binary variable	Cyclin D1, CCNE, p27, p21, p53	Santa Cruz (NR)	Immunohistochemistry
Brunner et al. [14]	2008	Switzerland	99	NR	Ta–T4	Low and high	1.5% or 23%	OS/RFS	Survival curve	MTS	NeoMarkers (1 : 50)	Immunohistochemistry
Friedrich et al. [15]	2001	Germany	40	NR	Ta–T1	G1–G3	5%	RFS/CP	Survival curve	LOH	Pharmingen (1 : 100)	Immunohistochemistry
Korkolopoulou et al. [16]	2001	Greece	23	72 (35–92)	T3-T4	Low and high	5%	OS/CP	Survival curve	P53	Santa Cruz (1 : 100)	Immunohistochemistry
Niehans et al. [17]	1999	USA	78	64.7 (48–82)	T1–T4	G2–G4	Score = 4	DSS/CP	Survival curve	P53, PRB, cyclin D1	Pharmingen (1 : 400)	Immunohistochemistry
Røtterud et al. [18]	2002	Norway	59	64 (42–75)	T2–T4	G2-G3	Score = 3	CSS/CP	Survival curve	p21, p27	NeoMarkers (1 : 100)	Immunohistochemistry
Vallmanya Llena et al. [19]	2006	Spain	97	NR	Ta–T1	Low and high	15%	RFS/PFS/OS/CP	Survival curve	p53, p21	DakoCytomation (NR)	Immunohistochemistry
Sun et al. [20]	2000	China	60	NR	Tis–T4	G1–G3	Score = 4	OS	Survival curve	PRb	Santa Cruz (1 : 100)	Immunohistochemistry
Santos et al. [21]	2003	Portugal	56 (40/16)	70 (43–83)	Ta–T1	G1-G2	20%	RFS/CP	Binary variable	p27, pRb, p53, Ki-67	Pharmingen (1 : 500)	Immunohistochemistry
Yin et al. [22]	2008	USA	18	NR	T1–T4	Low and high	Score = 4	CP	Binary variable	9p21	Pharmingen (1 : 250)	Immunohistochemistry
Primdahl et al. [23]	2002	Denmark	69 (55/14)	71 (42–83)	Ta–T4	G1–G4	Score = 4	CP	Binary variable	Rb, p27, p21, L-myc	NeoMarkers (1 : 50)	Immunohistochemistry
Jin et al. [24]	2006	USA	39 (25/14)	65 (42–84)	T2–T4	G1–G4	10%	CP	Binary variable	P53, pRB	NR (1 : 50)	Immunohistochemistry
Tzai et al. [25]	2004	China (Taiwan)	65 (44/21)	61.5 (41–84)	T2–T4	G2-G3	Score = 4	CP	Binary variable	P53, pRB	Santa Cruz (1 : 20)	Immunohistochemistry
Jin et al. [26]	2004	China	62 (32/30)	61 (18–80)	Tis–T4	G1–G3	OC	CP	Binary variable	Cyclin D1, PCNA	NR	Immunohistochemistry
Fu and Li [27]	2011	China	50 (39/11)	59.3 (32–81)	Tis–T4	G1–G3	10%	CP	Binary variable	E-cadherin	NR	Immunohistochemistry
Shi et al. [28]	2001	China	62 (52/10)	58.5 (22–87)	Tis–T4	G1–G3	Score = 3	RFS/CP	Binary variable	PCNA	Zymed (1 : 50)	Immunohistochemistry
Wang [29]	2001	China	49 (39/10)	61 (22–89)	NR	G1–G3	10%	RFS/CP	Binary variable	NR	NR	Immunohistochemistry
Miao [30]	1999	China	50	NR	Tis–T4	G1–G3	OC	RFS/OS/CP	Binary variable	Cyclin D1	Santa Cruz (1 : 100)	Immunohistochemistry
Shi et al. [31]	2003	China	82 (65/17)	58.7 (24–72)	Tis–T4	G1–G3	OC	RFS/CP	Binary variable	Cyclin D1	NR	Immunohistochemistry
Yang [32]	2005	China	69 (62/7)	61 (42–75)	Tis–T4	G1–G3	5%	RFS/CP	Binary variable	P27/nm23	NR	Immunohistochemistry
Wang et al. [33]	2013	China	45 (30/15)	65 (38–80)	NR	H/L	5%	RFS/CP	Binary variable	PTEN/P53	NR	Immunohistochemistry
Leng et al. [34]	2000	China	51 (43/8)	53.4 (28–72)	Tis–T3	G1–G3	OC	RFS/OS/CP	Binary variable	bcl-2	Santa Cruz (1 : 50)	Immunohistochemistry
Bai and Xiong [35]	2014	China	65 (50/15)	(57.7 ± 8.2)	Tis–T4	H/L	5%	CP	Binary variable	mfn2	Zymed (NR)	Immunohistochemistry
Wang et al. [36]	2000	China	75 (62/13)	58.5 (24–81)	Tis–T4	G1–G3	OC	CP	Binary variable	c-erbB-2, p53	Maxim (1 : 50)	Immunohistochemistry
Wang et al. [37]	2006	China	55 (35/20)	63 (24–75)	Tis–T4	G1–G3	10%	CP	Binary variable	hTERT, cyclin D1, RB	NR	Immunohistochemistry
Lu et al. [38]	2008	China	40 (30/10)	54.2 (37–79)	Tis–T4	G1–G3	10%	CP	Binary variable	p53, PCNA	NR (1 : 50)	Immunohistochemistry
Xie et al. [39]	2003	China	72 (56/16)	NR (29–78)	Tis–T4	G1–G3	5%	RFS/CP	Binary variable	Rb, cyclin D1	Zymed (1 : 50)	Immunohistochemistry
Qiu et al. [40]	2006	China	53 (46/7)	61 (25–83)	Tis–T4	G1–G3	15%	CP	Binary variable	NR	NR	Immunohistochemistry
Rebouissou et al. [41]	2012	France	89	NR	Ta–T1	G1–G3	Score = 3	RFS/PFS	Survival curve	FGFR3	NR	FISH
Abat et al. [42]	2014	Turkey	34 (30/4)	NR	T1–T4	Low and high	OC	PFS	Reported	p53	NR	FISH

M: male; F: female; RFS: recurrence-free survival; OS: overall survival; PFS: progression-free survival; DSS: disease-specific survival; CSS: cancer-specific survival; CP: clinicopathological parameters; OC: other criteria; NR: not reported; No.^b^: number of patients.

**Table 2:** Main meta-analysis results of p16 expression in patients with bladder cancer.

Analysis	No.^a^ (No.^b^)	HR (95% CI)	*Z*	*P*	Model	Heterogeneity		Publication bias	
						*I* ^2^%	*P*_het_	Begg's *P*	Egger's *P*
*RFS*	18 (1032)	1.63 (1.36–1.94)	5.40	*P* < 0.001	F	42.6	0.029	0.405	0.246
Europe	8 (365)	1.90 (1.13–3.19)	2.43	*P* = 0.003	R	53.1	0.037	1.000	0.749
Asia	9 (547)	1.44 (1.15–1.81)	3.15	*P* = 0.002	F	30.7	0.173	0.348	0.020
America	1 (120)	1.58 (0.77–3.25)	1.24	*P* = 0.214	F	0.0	/	/	/
Ta–T1	8 (430)	1.96 (1.23–3.14)	2.82	*P* = 0.005	R	55.5	0.028	0.711	0.916
Ta–T4	10 (602)	1.41 (1.12–1.77)	2.96	*P* = 0.003	F	10.2	0.348	1.000	0.062
G1-G2	2 (75)	4.12 (2.48–6.83)	5.49	*P* < 0.001	F	0.0	0.924	1.000	/
G1–G3	14 (762)	1.44 (1.18–1.75)	3.50	*P* < 0.001	F	11.9	0.323	0.584	0.031
G2-G3	2 (95)	1.37 (0.78–2.42)	1.10	*P* = 0.273	F	0.0	0.802	1.000	/
Cut-off value (≤10%)	13 (741)	1.83 (1.34–2.51)	3.79	*P* < 0.001	R	54.8	0.009	0.583	0.297
Cut-off value (>10%)	5 (291)	1.34 (0.86–2.09)	1.28	*P* = 0.200	F	0.0	0.701	0.462	0.166
*OS*	9 (425)	1.70 (1.16–2.50)	2.71	*P* = 0.007	F	0.0	0.584	0.602	0.165
Europe	4 (167)	2.54 (1.05–6.15)	2.07	*P* = 0.039	F	27.0	0.250	1.000	0.289
Asia	4 (208)	1.61 (0.97–2.66)	1.85	*P* = 0.065	F	0.0	0.703	0.734	0.166
America	1 (50)	1.41 (0.62–3.19)	0.83	*P* = 0.409	F	0.0	/	/	/
Ta–T4	5 (230)	1.59 (0.98–2.60)	1.87	*P* = 0.061	F	3.1	0.389	1.000	0.232
Ta–T1	2 (122)	1.57 (0.32–7.75)	0.55	*P* = 0.579	F	0.0	0.750	1.000	/
T2–T4	2 (73)	1.96 (0.99–3.88)	1.94	*P* = 0.053	R	52.1	0.148	1.000	/
G1–G3	7 (353)	1.82 (1.16–2.84)	2.62	*P* = 0.009	F	3.9	0.397	/	/
L	1 (22)	1.41 (0.18–10.90)	0.33	*P* = 0.742	F	/	/	/	/
H	1 (50)	1.41 (0.62–3.19)	0.83	*P* = 0.409	F	/	/	/	/
Cut-off value (≤10%)	7 (278)	1.83 (1.17–2.86)	2.63	*P* = 0.008	F	3.2	0.402	0.764	0.185
Cut-off value (>10%)	2 (147)	1.40 (0.66–2.96)	0.87	*P* = 0.384	F	0.0	0.951	1.000	/
*PFS*	8 (470)	2.18 (1.37–3.48)	3.28	*P* < 0.001	F	26.3	0.219	0.174	0.325
IHC	6 (347)	1.84 (1.13–3.01)	2.44	*P* = 0.015	F	0.0	0.487	1.000	0.754
FISH	2 (123)	11.28 (2.45–51.83)	3.11	*P* = 0.002	F	0.0	0.718	1.000	/
Europe	5 (297)	2.09 (1.21–3.63)	2.62	*P* = 0.009	F	0.0	0.484	1.000	0.607
America	1 (50)	1.14 (0.39–3.31)	0.24	*P* = 0.810	F	/	/	/	/
Ta–T1	5 (297)	2.09 (1.21–3.63)	2.62	*P* = 0.009	F	0.0	0.484	1.000	0.607
T2–T4	1 (50)	1.14 (0.39–3.31)	0.24	*P* = 0.810	F	/	/	/	/
G1–G3	5 (297)	2.09 (1.21–3.63)	2.62	*P* = 0.009	F	0.0	0.484	1.000	0.607
H	1 (50)	1.14 (0.39–3.31)	0.24	*P* = 0.810	F	/	/	/	/
Cut-off value (≤10%)	4 (200)	2.61 (1.42–4.77)	3.10	*P* = 0.002	F	0.0	0.932	1.000	0.746
Cut-off value (>10%)	2 (147)	0.95 (0.41–2.18)	0.13	*P* = 0.896	F	0.0	0.579	1.000	/
*DSS/CSS*	3 (187)	1.52 (0.85–2.71)	1.42	*P* = 0.155	F	0.0	0.825	0.296	0.517

Clinicopathological parameters		OR (95% CI)							

Stage (T2–T4 versus Ta–T1)	19 (1231)	3.13 (2.42–4.06)	8.63	*P* < 0.001	F	1.4	0.440	0.529	0.377
Asia	14 (878)	3.41 (2.51–4.64)	7.87	*P* < 0.001	F	0.0	0.800	0.661	0.650
Europe	3 (277)	3.17 (1.79–5.60)	3.96	*P* < 0.001	F	63.7	0.064	1.000	0.994
America	2 (76)	1.15 (0.41–3.20)	0.26	*P* = 0.796	F	0.0	0.604	1.000	/
Stage (T1 versus Ta)	5 (374)	1.55 (0.87–2.76)	1.50	*P* = 0.134	F	40.5	0.152	0.806	0.402
Grade (G3 versus G1-2)	20 (1291)	3.33 (2.51–4.42)	8.32	*P* < 0.001	F	0.0	0.519	0.206	0.805
Asia	15 (895)	3.36 (2.44–4.63)	7.41	*P* < 0.001	F	18.6	0.246	/	/
Europe	3 (196)	2.62 (1.23–5.57)	2.50	*P* = 0.013	F	0.0	0.984	/	/
America	2 (200)	4.51 (1.61–12.61)	2.87	*P* = 0.004	F	0.0	0.659	/	/
Grade (H versus L)	8 (688)	1.20 (0.62–2.33)	0.55	*P* = 0.580	R	61.8	0.011	0.063	0.080
Lymph node metastasis (yes versus no)	5 (319)	2.20 (1.26–3.83)	2.77	*P* = 0.006	F	27.2	0.240	1.000	0.487
Muscle Invasive (yes versus no)	4 (248)	2.18 (0.72–6.62)	1.38	*P* = 0.167	R	71.8	0.014	0.497	0.998
Number of tumors (multiple versus single)	2 (166)	1.11 (0.43–2.85)	0.22	*P* = 0.823	F	0.0	0.984	1.000	/
Tumor size (>3 versus ≤3)	2 (193)	2.93 (0.40–21.36)	1.06	*P* = 0.289	R	79.2	0.028	1.000	/

RFS: recurrence-free survival; OS: overall survival; PFS: progression-free survival; DSS: disease-specific survival; CSS: cancer-specific survival; HR: hazard ratio; OR: odds ratio; CI: confidence interval; No.^a^: number of studies; No.^b^: number of patients; *P*_het_: *P* for the heterogeneity; F: fixed-effect model; R: random-effect model; L: low grade; H: high grade.

**Table 3:** Relationship between low expression of p16 and other prognostic factors in patients with bladder cancer.

Author	Year	Nation	No.^b^ (M/F)	Age	Stage	Grade	Cut-off value	Other related biomarkers	Measuring method	Antibody source (dilution)	Outcome	*P* value		
Jin et al. [24]	2006	USA	39 (25/14)	65 (42–84)	T2–T4	G1–G4	10%	P53, pRB	Immunohistochemistry	NR (1 : 50)	OS/PFS (2-year survival)	0.001	<0.001	
Tzai et al. [25]	2004	China (Taiwan)	65 (44/21)	61.5 (41–84)	T2–T4	G2-G3	Score = 4	P53, pRB	Immunohistochemistry	Santa Cruz (1 : 20)	PFS/DSS	0.74	0.49	
Yurakh et al. [6]	2006	Spain	55	NR	Ta–T4	G1–G3	10%	9p21 (P14, P15, P16)	Immunohistochemistry	Santa Cruz (1 : 500)	RFS/OS/PFS (3-year survival)	0.31	0.022	0.012

M: male; F: female; RFS: recurrence-free survival; OS: overall survival; PFS: progression-free survival; DSS: disease-specific survival; NR: not reported; No.^b^: number of patients.

## Abbreviations

RFS: Recurrence-free survival
OS: Overall survival
PFS: Progression-free survival
DSS: Disease-specific survival
CSS: Cancer-specific survival
HR: Hazard ratio
OR: Odds ratio
CI: Confidence interval.
